# Uterine Carcinosarcoma: Adaptation to New FIGO 2023 Staging System Through Clinical Profile and Oncologic Outcomes

**DOI:** 10.3390/jcm14072299

**Published:** 2025-03-27

**Authors:** Saliha Sağnıç, Serap Fırtına Tuncer, Elif Iltar, Fatma Ceren Güner, Hasan Aykut Tuncer, Selen Doğan, Tayup Şimşek

**Affiliations:** 1 Division of Gynecologic Oncology, Department of Gynecology Obstetrics, Akdeniz University, Antalya 07070, Turkey; dktr_elif@hotmail.com (E.I.); fcereng@gmail.com (F.C.G.); aykuttuncer@hotmail.com (H.A.T.); drsalben@hotmail.com (S.D.); tsimsek@akdeniz.edu.tr (T.Ş.); 2 Department of Obstetrics and Gynecology, Antalya Education & Research Hospital, Antalya 07100, Turkey; drserap.firtina@hotmail.com

**Keywords:** FIGO staging, prognostic precision, stage shift, uterine carcinosarcoma

## Abstract

**Objective:** We aimed to analyze the impact of stage shifts on disease-free survival and overall survival in patients with uterine carcinosarcoma stratified based on FIGO 2009 and 2023 staging systems. **Materials and Methods:** A total of forty-five patients diagnosed with uterine carcinosarcoma between 2010 and 2024 were included in the study. Patients were classified and analyzed according to both the 2009 and the revised 2023 FIGO staging systems to evaluate the impact of the updated staging criteria on oncologic outcomes. The median disease-free (DFS) and overall survival (OS) rates were calculated and compared when stage shifts occurred. **Results**: A total of 17 upstage shifts (37.7%) occurred between the 2009 and 2023 FIGO staging system. All patients with upstage shifts were stage I patients categorized according to the FIGO 2009 classification. Restaging from the FIGO 2009 to the FIGO 2023 criteria resulted in a reduction in the number of stage I cases and an increase in the number of stage II cases. The two main factors leading to upstage were serous histology and LVSI positivity. The 5-year DFS and OS rates for stage I disease were 80% and 75%, respectively, according to the 2009 FIGO staging system, whereas the 2023 FIGO staging system demonstrated significantly higher rates of 100% for both DFS and OS. In stage II patients, the 5-year DFS and OS rates were 33.5% and 33.7%, respectively, according to the 2009 FIGO staging system, while the 2023 FIGO staging system demonstrated higher rates of 58.8% for DFS and 65% for OS. **Conclusions:** The revised FIGO 2023 staging system has better performance in predicting disease prognosis than the previous version.

## 1. Introduction

Uterine carcinosarcoma (UC), previously classified as a malignant mixed Müllerian tumor, is a rare, highly aggressive, and biphasic neoplasm characterized histologically by the presence of dual malignant components: epithelial (carcinomatous) and mesenchymal (sarcomatous) elements. The epithelial component is typically poorly differentiated, exhibiting features such as endometrioid, serous, or clear cell differentiation, or, in some cases, undifferentiated characteristics. The sarcomatous components are widely regarded as dedifferentiated derivatives of an initial carcinomatous progenitor. These components may consist of either homologous mesenchymal tumors, which resemble cell types naturally present in the uterus, or heterologous mesenchymal tumors, which exhibit differentiation into cell types not typically found in the uterus [1].

The incidence of this tumor in the United States varies between 5.1 and 6.9 cases per 1,000,000 individuals annually [2,3]. It represents approximately 5% of all uterine malignancies and accounts for nearly 20% of non-endometrioid endometrial cancers [4,5]. The median overall survival is under 2 years, with a 5-year overall survival rate of less than 30%. Specifically, the survival rates are approximately 50% for early-stage disease and 20% for advanced-stage disease. Even among patients diagnosed at an early stage, the 5-year recurrence rate is 45%, and the 5-year mortality rate related to the disease is 50% [5,6,7,8]. UC predominantly affects older, postmenopausal women and is associated with a significantly poorer prognosis compared to high-grade endometrial carcinoma [9,10]. The management of uterine carcinosarcomas continues to present significant challenges, primarily due to the rarity of the disease and its pronounced biological heterogeneity [11]. The majority of patients typically present with postmenopausal bleeding as the primary clinical symptom [12]. Comprehensive surgical staging to evaluate tumor dissemination, followed by systemic therapy for patients with both early- and advanced-stage disease, is essential in the management of UC. However, the role of postoperative radiation therapy remains uncertain and requires further investigation [13].

Carcinosarcoma is currently staged according to the endometrial carcinoma staging system [14], as its clinical behavior is predominantly driven by the carcinomatous component.

Accurate staging is a fundamental principle in guiding optimal treatment strategies, as tumor stage represents the most critical prognostic factor for both disease-free survival (DFS) and overall survival (OS) [11]. The FIGO (International Federation of Gynecology and Obstetrics) surgical staging system for endometrial cancer was revised in 2023 to incorporate new pathological and molecular features, which are now critically integrated into the staging process to enhance the accuracy of prognosis and treatment planning for patients. The revised FIGO staging system for endometrial cancer has evolved into a comprehensive framework that integrates surgical, pathological, and molecular criteria, reflecting a more holistic approach to accurately staging the disease and predicting patient outcomes [15]. Although the substages within the revised FIGO staging system may be challenging to memorize, it is widely advocated that this updated system provides a more precise prediction of prognosis for patients with endometrial cancer compared to the previous staging framework. However, the reliability and accuracy of the revised FIGO 2023 staging system in survival analysis have not yet been sufficiently validated, necessitating further research and clinical studies to confirm its prognostic utility.

The aim of our study was to compare the oncologic outcomes of patients with uterine carcinosarcoma staged according to the 2009 FIGO staging system and the revised 2023 FIGO staging system. Additionally, we aimed to provide a brief review of the clinicopathological characteristics of uterine carcinosarcomas treated at our institution.

## 2. Materials and Methods

We conducted a retrospective cohort analysis of patients diagnosed with uterine carcinosarcoma (UC) who underwent primary treatment at the Department of Gynecological Oncology, Akdeniz University Faculty of Medicine, Antalya, Turkey, between January 2010 and January 2024. The diagnosis of UC was confirmed through a combination of pre-operative biopsy specimens, hysterectomy samples, paracentesis, and liver biopsy, as clinically indicated. Informed consent was obtained from each subject or their first-degree relatives (for the deceased ones). Patients who were eligible for analysis were followed until May 2024.

The patients’ detailed clinical data at initial diagnosis and tumor clinicopathologic features were retrieved from paper and electronic medical records. Data on the stage according to the International Federation of Gynecology and Obstetrics (FIGO 2009 and 2023) staging classification [16,17], oncological outcome, overall survival (OS), and disease-free survival (DFS) were abstracted. Patients with ovarian, fallopian tube, and cervical carcinosarcoma, carcinosarcoma originating from non-gynecologic primary sites, second primary uterine carcinosarcoma after radiation therapy for cervical cancer, those with insufficient data or lack of attendance to follow-up, and those with endometrial metastasis originating from other gynecologic primary sites were excluded from the study. Following the implementation of the exclusion criteria, a total of 45 patients who met the inclusion criteria were identified and subsequently enrolled in the study within the designated time frame.

Patients were classified according to both the previous 2009 FIGO staging system and the revised 2023 FIGO staging system, and substage shifts were systematically documented and analyzed. Upstaging was defined as the reclassification of the cancer stage into a higher group if it meets the criteria of the new FIGO 2023 staging system. Downstaging was defined as shifting to a lower group according to the new classification system.

Endometrioid adenocarcinoma grades 1 and 2 were categorized as non-aggressive tumors, whereas endometrioid carcinoma grade 3, serous carcinoma, clear cell carcinoma, and undifferentiated carcinoma were classified as aggressive tumor types. Patients with mixed tumor types were staged according to the most aggressive histological component present in the tumor. Since the previous pathology reports did not explicitly differentiate between focal or extensive lymphovascular space invasion (LVSI), all patients with documented LVSI in their reports were classified as LVSI-positive (extensive invasion) for the purposes of this study.

Imaging modalities, including computed tomography (CT), magnetic resonance imaging (MRI), and positron emission tomography (PET), were utilized for the detection of disease relapse. OS was defined as the time from initial diagnosis to death. DFS was defined as the interval between the date of remission and the date of the first recurrence detected.

Statistical analysis was performed using SPSS Statistics version 23 (IBM Corp. Released 2015. IBM SPSS Statistics for Windows, Version 23.0. Armonk, NY, USA: IBM Corp.). Patient characteristics were summarized by descriptive statistics. The mean, standard deviation, median, minimum–maximum values, and frequencies were used for descriptive statistics, depending on the normality of the data. Data were expressed as mean ± standard deviation (SD), median, and *n* (%) where appropriate. Survival analysis was performed via Kaplan–Meier analysis. Survival curves were generated to evaluate DFS and OS according to FIGO 2009 and 2023 classifications, and the differences between the curves were interpreted. The median DFS and OS rates per substage were analyzed and compared between the previous and new staging systems. The Cox proportional hazard model was used to compare the prognostic accuracy of two staging systems in terms of DFS and OS. The Akaike information criterion (AIC), the Bayesian information criterion (BIC), Harrell’s concordance index (C-index), and the likelihood ratio test were utilized to evaluate the ability of the staging systems to discriminate between patients with longer versus shorter DFS and OS. A lower AIC or BIC value indicates a better model fit. *p*-values less or equal to 0.025 were considered significant.

## 3. Results

A total of 45 patients were included in the present study. Baseline demographic, clinical, and obstetric characteristics, tumor clinicopathologic features, and FIGO stage distribution of the patients are shown in Table 1.

The mean age at presentation was 61.8 years (range 42–83 years). The most prevalent histological type of the epithelial malignant component was serous carcinoma (60%), followed by endometrioid carcinoma (33.3%). Given that the majority of patients with malignant epithelial components of endometrioid type were classified as grade 3, this resulted in a cohort in which 84.4% exhibited aggressive histologic subtypes. LVSI was noticed in 44.4% of patients and twenty-seven (60%) women were positive for p53 immunohistochemistry.

The median follow-up period for the cohort was 44.9 months (range: 0.43–193.3 months), with a median overall survival of 52 months (95% CI: 2.9–101.1). The disease progressed in 13.3% of the cases (Table 2).

**Figure 1:**
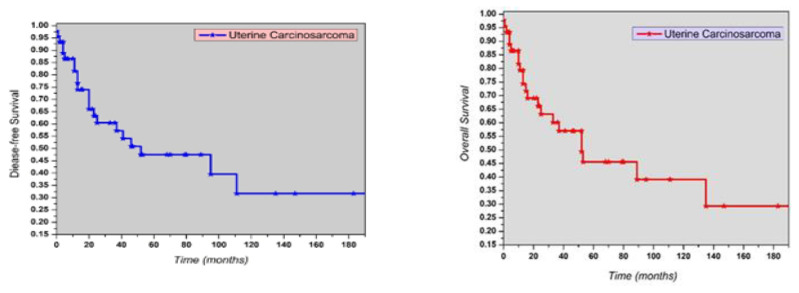
Kaplan–Meier curves for disease-free survival (DFS) and overall survival (OS) in women with uterine carcinosarcoma.

Moreover, 25 out of 45 (48.8%) patients died of the disease. Including all FIGO stages, the 5-year DFS and OS rates were 47.4% and 45.56%, respectively (Figure 1).

**Figure 2:**
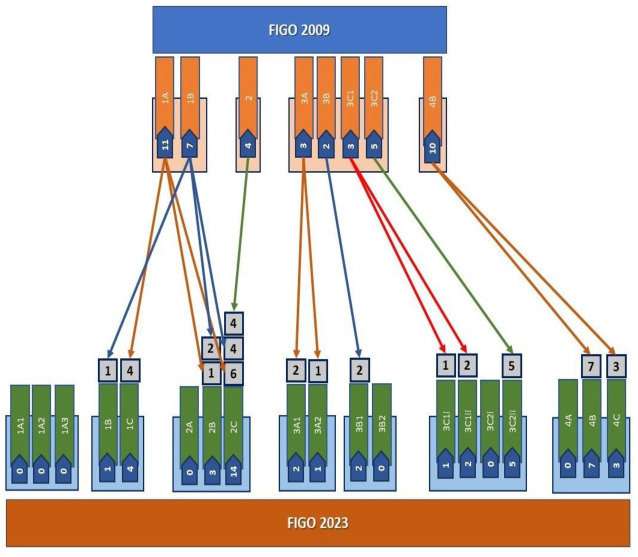
Stage shifts in study cohort of 45 UC patients according to 2009 and 2023 FIGO staging systems without molecular classification. Each colored arrow represents the reclassification of patient subgroups from the 2009 staging system to the revised 2023 system subgroups.

Forty percent (*n*: 18) of the patients were clustered in stage I, 8.9% (*n:* 4) in stage II, 13 (28.9%) in stage III, and 10 (22.2%) in stage IV according to the 2009 FIGO staging system. All cases were retrospectively restaged according to the newly adopted FIGO 2023 surgical staging criteria, revealing the following distribution: 11.1% (*n* = 5) of patients were classified as stage I, 37.8% (*n* = 17) as stage II, 28.9% (*n* = 13) as stage III, and 22.2% (*n* = 10) as stage IV (Figure 2).

According to the new 2023 staging system, 37 out of 45 patients were moved to subgroup stages. A total of 17 upstage shifts (37.7%) occurred between the 2009 and 2023 FIGO staging system. No downstage shifts were noted among the groups. All patients with upstage shifts were stage I patients categorized according to the FIGO 2009 classification. Restaging from FIGO 2009 to FIGO 2023 criteria decreased the number of stage I cases from 18 (40%) to 5 (11.1%). Restaging from FIGO 2009 to FIGO 2023 criteria increased the number of stage II cases from 4 (8.9%) to 17 (37.8%). The upshifts occurred from stages IA to stage IIB (*n*: 1) and to stage IIC (*n*: 6). Two patients in stage IB were upstaged to stage IIB and four patients to stage IIC. Twenty-four patients (53.3%) were shifted to subgroups of their current stage. The number of patients in stages 3 and 4 did not change. Eight patients (17.7%) maintained their current stage (Figure 2). The histological subtype for upstaged cases was mainly serous (66.6%) and high-grade (77.7%). Stage shift occurred due to LVSI positivity in 16.6% of patients. Apart from IA and IB, no upstage was observed in other advanced stages.

The median relapse-free survival was 71.5 months for stage I patients and 29.5 months for stage II patients when staged according to the FIGO 2009 staging system. In contrast, when restaged using the FIGO 2023 staging system, the median relapse-free survival was 112.6 months for stage I patients and 49.5 months for stage II patients. The median OS was 75 months for stage I patients and 43.6 months for stage II patients when staged according to the FIGO 2009 staging system. In contrast, when restaged using the FIGO 2023 staging system, the median OS was 112.6 months for stage I patients and 56.5 months for stage II patients. There was no difference between the 2009 and 2023 staging systems in terms of DFS and OS of patients in stages III and IV (Table 3).

**Figure 3:**
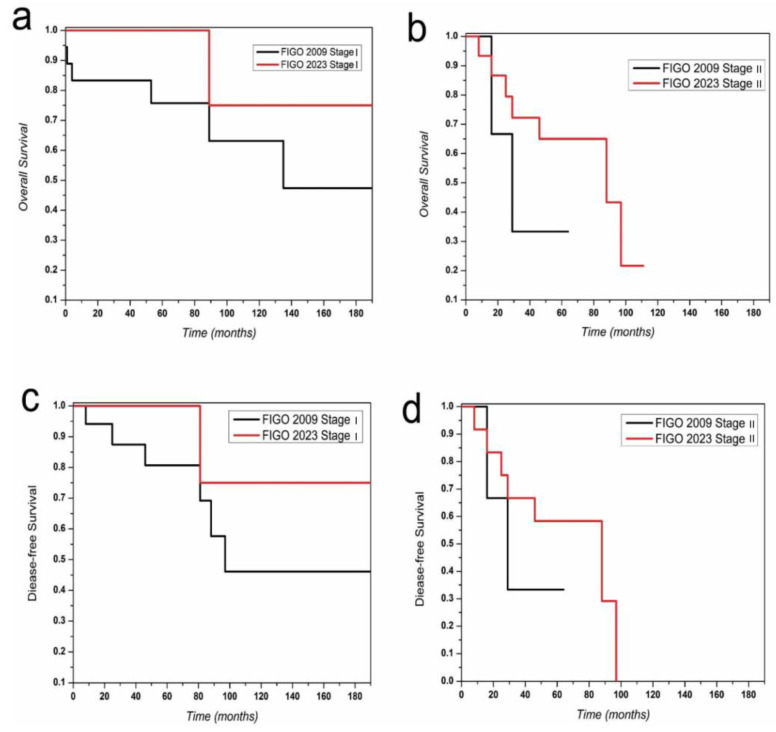
(**a**) Kaplan–Meier curves for overall survival (OS) in stage I uterine carcinosarcoma cancer patients according to 2009 and 2023 FIGO staging system. (**b**) Kaplan–Meier curves for overall survival (OS) in stage II uterine carcinosarcoma cancer patients according to 2009 and 2023 FIGO staging system. (**c**) Kaplan–Meier curves for disease-free survival (DFS) in stage I uterine carcinosarcoma cancer patients according to 2009 and 2023 FIGO staging system. (**d**) Kaplan–Meier curves for disease-free survival (DFS) in stage II uterine carcinosarcoma cancer patients according to 2009 and 2023 FIGO staging system.

DFS and OS estimates of stages I and II are shown in Figure 3 according to the FIGO 2009 and 2023 staging criteria.

The 5-year DFS and OS rates for stage I disease were 80% and 75%, respectively, according to the 2009 FIGO staging system, whereas the 2023 FIGO staging system demonstrated significantly improved rates of 100% for both DFS and OS. Similarly, for stage II patients, the 5-year DFS and OS rates calculated using the 2009 FIGO staging system were lower than those derived from the 2023 staging system, with rates of 33.5% and 33.7% versus 58.8% and 65%, respectively. The 2023 FIGO staging system for stage I had significantly lower AIC and BIC values for predicting endpoint OS compared to the 2009 FIGO staging system (375.06 vs. 744.6 and 376.90 vs. 745.80, respectively). The 2023 FIGO staging system for stage I had significantly lower AIC and BIC values for predicting endpoint DFS compared to the 2009 FIGO staging system (253.50 vs. 744.08 and 253.76 vs. 735.10, respectively).

The 2023 FIGO staging system for stage II had significantly lower AIC and BIC values for predicting endpoint OS compared to the 2009 FIGO staging system (161.62 vs. 649.43 and 160.40 and 650.54, respectively). The 2023 FIGO staging system for stage II had significantly lower AIC and BIC values for predicting endpoint DFS compared to the 2009 FIGO staging system (161.32 vs. 529.67 and 160.32 vs. 530.78, respectively). The C-index was 0.87 and 0.77, respectively, for the 2023 and 2009 FIGO staging systems to predict the endpoint of OS. The likelihood ratio test comparing the model fits was significant (*p* = 0.020). The C-index was 0.79 and 0.71, respectively, for the 2023 and 2009 FIGO staging systems to predict the endpoint of DFS. The likelihood ratio test comparing the model fits was significant (*p* = 0.016). The likelihood ratio comparing the two models showed a significantly better fit for the 2023 FIGO staging system (*p*-value = 0.0158).

## 4. Discussion

To the best of our knowledge, this study represents the first comprehensive analysis to evaluate the impact of stage shift on DFS and overall survival OS in patients with uterine carcinosarcoma, stratified according to both the FIGO 2009 and the revised FIGO 2023 staging criteria. Recent studies have categorized patients with endometrial carcinoma according to both the previous FIGO staging system and the revised version, analyzed stage shifts, and compared DFS and OS rates to assess the impact of updated staging criteria on oncologic outcomes [18,19]. However, patients with carcinosarcoma were underrepresented in these studies. Our findings suggest that the newly revised FIGO 2023 staging system has the potential to improve prognostic prediction, particularly in patients with early-stage UC. However, its utility in advanced-stage disease remains to be further elucidated. The oncologic prognosis of patients with advanced-stage UC could not be adequately compared due to the limited number of cases and the absence of stage shift in this subgroup, as the staging criteria for advanced disease remained unchanged between the FIGO 2009 and 2023 systems. Similarly, in the study by Schwameis et al. [18], the impact of the 2023 FIGO staging system on the prognosis of patients with advanced-stage disease could not be clearly determined due to the limited number of cases in this category. However, a validation study involving a large cohort of patients with advanced endometrial cancer demonstrated that the newly revised FIGO 2023 staging system revealed significant differences in survival rates, highlighting its potential utility in prognostic stratification for advanced-stage disease. The authors not only demonstrated that stage IIIB1-2 is associated with a higher mortality rate compared to stage IIIA1-2 but also provided evidence that patients downstaged from IIIA1-2 to IA3 under the 2023 FIGO staging system exhibit a significantly lower 5-year cancer-related mortality rate [20]. In contrast to these studies, our analysis focused exclusively on patients with UC, a subtype associated with significantly worse survival outcomes compared to endometrial carcinoma.

It is noteworthy that more than one-third of our entire cohort experienced stage shift, and all of these patients were initially classified as stage I according to the previous FIGO 2009 staging system. When stage shifts were analyzed, it was observed that all shifts resulted in upstaging, with no patients being downstaged to a lower subgroup. This observation is attributed to the fact that the majority of UC exhibit a serous epithelial component, which is classified as an aggressive histological subtype, thereby influencing the upstaging under the revised FIGO 2023 criteria. The second significant factor contributing to these upshifts was the presence of LVSI, even when combined with a non-aggressive histological subtype, a feature that was not incorporated into the 2009 FIGO staging system but is now recognized in the 2023 revision.

According to the FIGO 2009 staging system, tumors should not be upstaged solely on the basis of the presence of LVSI in the absence of myometrial involvement, and such cases are classified as FIGO stage IA. Extensive research has demonstrated that LVSI serves as an independent prognostic factor in endometrial carcinomas [21,22,23,24,25,26]. Previous studies have recommended that patients exhibiting LVSI in the absence of myometrial invasion should not be upstaged based solely on this criterion. Instead, the presence of LVSI should primarily be taken into consideration when determining the necessity of adjuvant treatment [27,28]. Since 2023, with the prognostic significance of LVSI being well established, both the staging and treatment strategies for patients were revised to incorporate LVSI positivity as a decisive factor, independent of other pathological features. Under the revised staging system, these patients are now classified as FIGO stage IIB due to the presence of LVSI, and they are subsequently managed with the most appropriate treatment modality tailored to their updated stage. Consequently, the revision of the previous staging system represents a more effective model for predicting prognosis, as it not only assigns aggressive histologic types to higher stages but also incorporates LVSI as a critical factor in determining the most appropriate treatment modalities for patients. Therefore, it is imperative for pathologists to meticulously evaluate and accurately identify both the histotype of the epithelial component and the presence of LVSI prior to establishing a final diagnosis.

The majority of patients diagnosed with endometrial cancer present with stage I disease, and a significant proportion of these individuals achieve complete remission and are effectively cured. However, this patient category does not represent a homogeneous population when stratified according to the FIGO 2009 staging system. This group comprises a heterogeneous cohort of patients characterized by diverse histologic types of epithelial components, all of whom exhibit no evidence of dissemination beyond the uterus. Despite being diagnosed at an early stage, a subset of these patients may experience disease recurrence and ultimately die. The recurrence and mortality in early-stage disease stand in contrast to the well-established principle that stage is the most significant prognostic factor in cancer. Therefore, the revised staging system was designed to serve as an optimal framework for achieving homogeneous stratification, accurate prognostication, and tailored treatment recommendations for this patient population. In this revised model, high-grade histologic types were reclassified into higher stages, leading to enhanced accuracy in survival prediction. Previous studies have demonstrated that histologic typing possesses a superior capacity for predicting survival outcomes [29]. The primary limitation of the revised staging system lies in its complexity, as each stage encompasses numerous subgroups, rendering it impractical to memorize and necessitating the expertise of highly experienced pathologists for accurate implementation.

The implementation of comprehensive molecular classification—categorizing endometrial cancers into distinct subgroups such as POLEmut, MMRd, NSMP, and p53abn—is strongly recommended for all cases of endometrial carcinoma to enhance diagnostic precision, prognostic stratification, and therapeutic decision-making. High-grade endometrial carcinomas (e.g., uterine carcinosarcoma) derive the greatest benefit from the application of molecular classification, since traditional histopathological assessment may be insufficient for precise risk stratification. When molecular classification identifies TP53 mutation or POLEmut (POLE-mutated) status in stage I and II endometrial cancers, it can lead to significant adjustments in disease staging, either upstaging or downstaging the tumor. No significant alterations are observed in the molecular staging of patients diagnosed with stage III and stage IV disease [17]. Moreover, molecular profiling within this high-grade cohort enables the differentiation of a subgroup with an excellent prognosis (POLE-mutated in early-stage disease, irrespective of grade) from a subgroup associated with a poor prognosis (p53 abnormal). Current data suggest that carcinomas classified within the POLE-mutated subgroup may derive significant benefit from the de-escalation of postoperative adjuvant therapy, given their consistently favorable outcomes [30]. In contrast, tumors characterized by p53 abnormalities (p53abn) are associated with a markedly poorer prognosis, indicating that intensified therapeutic approaches may be warranted to improve clinical outcomes in this subgroup [31,32]. The presence of specific molecular alterations may significantly influence decisions regarding adjuvant and systemic treatment strategies [33,34]. Consequently, these molecular profiles have the potential to impact OS and DFS.

We were unable to evaluate patient survival based on the molecular classification of the tumor due to the unavailability of relevant data. Our analysis revealed that 60% of the patient population exhibited immunohistochemical p53 positivity, with 31.1% of these cases identified within stages I–II. The majority of carcinosarcomas (70%) exhibit p53 abnormalities, which are associated with a poor prognosis, consequently necessitating treatment with chemoradiotherapy [1,35]. p53 immunohistochemistry (IHC) is a widely used surrogate marker reflecting the mutational status of TP53 [36]. Determining p53 status through IHC represents a rapid, cost-effective, and reliable diagnostic approach that can be integrated into the clinical management of carcinosarcoma. This method may be particularly advantageous over next-generation sequencing in low-resource settings due to its accessibility and affordability [37]. Molecular classification may hold limited utility for a small subset of these high-grade tumors, as adjuvant treatment is typically administered even to early-stage patients, distinguishing this approach from the management of endometrial cancer. The impact of the FIGO 2023 staging system on survival prediction could be further elucidated if molecular classification data were available for analysis. Despite this limitation, we demonstrated that the FIGO 2023 staging system outperforms its predecessor when evaluated solely based on surgical and clinicopathologic features. The subset of patients in our cohort exhibiting OS and DFS rates of 100% in stage I may potentially harbor pathogenic POLE mutations, as no relapses or deaths were observed during the follow-up period.

The primary limitations of this study include its retrospective single-center design, which introduces inherent biases, and the small sample size of the cohort, a consequence of the rarity of these tumors. These constraints hindered the ability to compare survival outcomes across substages and advanced stages within the FIGO 2009 and 2023 staging systems. Further studies involving larger cohorts of patients with advanced carcinosarcoma are necessary to provide more comprehensive insights and validate these findings. Data were collected over several years from a single institution. Additionally, we were unable to evaluate the impact of molecular characterization—such as POLE mutational analysis, mismatch repair protein status, and p53 status analysis—on survival outcomes in endometrial carcinosarcoma patients. This is due to the fact that such advanced analyses have not yet been implemented in low-income countries, primarily because of insufficient financial resources and support. Furthermore, patients classified as stage IIB under the 2023 staging system may be subject to over-staging, as the distinction between focal and substantial LVSI could not be adequately assessed due to insufficient data. If the pathology reports had provided more detailed information on LVSI, the survival outcomes for each stage within the study population might have been significantly different.

## 5. Conclusions

In conclusion, the revised FIGO 2023 staging system demonstrates greater reliability in predicting disease-free survival (DFS) and overall survival (OS), as evidenced by comprehensive statistical analysis. Nevertheless, the revised FIGO 2023 staging system is more complex and less practical to memorize in comparison to its earlier version. A meticulous evaluation of epithelial components and LVSI by pathologists is particularly critical for patients diagnosed at early stages, as some individuals are now reclassified into higher stages, resulting in altered prognostic outcomes compared to previous assessments.

## Figures and Tables

**Table 1:** Baseline demographic, clinical, and obstetric characteristics and tumor clinicopathologic features in patients with uterine carcinosarcoma.

	Uterine Carcinosarcoma (*n*: 45)
**Age (years), mean ± SD**	61.8 ± 8.8
**BMI (kg/m^2^), mean ± SD**	31.1 ± 5.05
**Gravidity/Parity, mean**	3.8/3.06
Multipar, *n* (%)	44 (97.8)
Nullipar, *n* (%)	1 (2.2)
**Mode of delivery, *n* (%)**	44 (97.8)
NVD	3 (6.7)
CS	41(91.1)
**Menopausal status, *n* (%)**	
Premenopausal	3 (6.7)
Postmenopausal	42 (93.3)
**Comorbidity, *n* (%)**	28 (62.2)
None	17 (37.8)
Diabetes mellitus	7 (15.6)
Arterial hypertension	15 (33.3)
CAD	4 (8.9)
**ECOG PS, *n* (%)**	
PS 0	9 (20.0)
PS 1	29 (64.4)
PS 2	4 (8.9)
PS 3	3 (6.7)
**ASA score, *n* (%)**	
1	3 (6.7)
2	38 (84.4)
3	4 (8.9)
**Symptomatology, *n* (%)**	
Postmenopausal bleeding	36 (80.0)
Pelvic pain	7 (15.6)
Abdominal distension	2 (4.4)
**Diagnostic methods, *n* (%)**	
Pipelle biopsy	10 (22.2)
Hysteroscopic biopsy	3 (6.7)
Hysteroctomy	15 (33.3)
D & C	14 (31.1)
Others *	3 (6.6)
**Epithelial component, histologic subtype, *n* (%)**	
Endometrioid	15 (33.3)
Serous	27 (60.0)
Undifferentiated	1 (2.2)
Mixed histology	2 (4.4)
Binary FIGO grading, *n* (%) **	
Low-grade	7 (15.6)
High-grade	38 (84.4)
**LVSI, *n* (%)**	
No	14 (31.1)
Yes	20 (44.4)
Missing	11 (24.4)
**Sarcomatous component histologic subtype, *n* (%)**	
LGESS	3 (6.7)
HGESS	11 (24.4)
Rhabdomyosarcoma	3 (6.7)
Osteosarcoma	3 (6.7)
Chondrosarcoma	17 (37.8)
Leiomyosarcoma	3 (6.7)
Undifferentiated uterine sarcoma	4 (8.9)
**Mesenchymal component, *n* (%)**	
Homologous	21 (46.7)
Heterologous	24 (53.3)
**FIGO 2009, *n* (%)**	
Stage I	18 (40.0)
Stage II	4 (8.9)
Stage III	13 (28.9)
Stage IV	10 (22.2)
**FIGO 2023, *n* (%)**	
Stage I	5 (11.1)
Stage II	17 (37.8)
Stage III	13 (28.9)
Stage IV	10 (22.2)

Note: SD, standard deviation; BMI, body mass index; NVD, normal vaginal delivery; CS, cesarean section; CAD, coronary artery disease; ECOG PS, Eastern Cooperative Oncology Group performance status; FIGO, International Federation of Gynecology and Obstetrics; ASA, American Society of Anesthesiologists; D&C, dilatation and curretage; LVSI, lymphovascular space invasion; LGESS, low-grade endometrial stromal sarcoma; HGESS, high-grade endometrial stromal sarcoma; * Others; liver biopsy, paracentesis. ** Low-grade is defined as grade 1 and 2 disease, high-grade refers to grade 3 carcinoma.

**Table 2:** Treatment modalities and oncological outcome in patients with UC.

	Uterine Carcinosarcoma (*n*: 45)
**Surgery type, *n* (%)**	
Exclusive surgery	38 (84.4)
Hysterectomy + BSO	4 (8.9)
None	3 (6.7)
**Adjuvant treatment, *n* (%)**	
CT	21 (46.7)
CRT	17 (37.8)
None	7 (15.5)
**Neoadjuvant treatment**, ***n* (%)**	
Yes	3 (6.7)
No	42 (93.3)
**Tumor size mm (mean ± SD)**	6.8 ± (3.7)
**Positive lymph nodes, *n* (%)**	10 (22.2)
**Number of CT cycles, median**	5.26 (2–9)
**Recurrence treatment, *n***	
Surgery and chemotherapy	3
Exclusive chemotherapy	9
Exclusive surgery	1
**Median follow-up (months)**	44.9 (0.43–193.3)
**Median time to recurrence (months)**	33.9
**Oncological outcome**	
Recurrence, *n* (%)	14 (31.1)
5-year DFS rate (%)	47.4
5-year OS rate (%)	45.56

Note: BSO, bilateral salping-ooferectomy; CT, chemotherapy; CRT, chemoradiotherapy; DFS, disease-free survival; OS, overall survival.

**Table 3:** Disease-free (DFS) and overall survival (OS) according to 2009 and 2023 FIGO staging system without molecular classification.

	FIGO 2009			FIGO 2023		
Stage	Patients, *n*	DFS, Median (Months)	OS Median (Months)	Patients, *n*	DFS Median (Months)	OS Median (Months)
**1**	18	**71.56**	**75.03**	5	**112.65**	**112.65**
**1A**	11	67.63	72.28	-	-	-
**1B**	7	**79.43**	**80.52**	1	**193.93**	**193.93**
**1C**	-	-	-	4	92.33	92.33
**2**	4	**29.52**	**43.68**	17	**49.59**	**56.59**
**2A**	-	-	-	-	-	-
**2B**	-	-	-	3	78.37	81.21
**2C**	-	-	-	14	43.41	51.31
**3**	13	19.85	25.11	13	19.85	25.11
**3A**	3	14.19	30.88	3	14.19	30.88
3A1	-	-	-	2	20.17	45.2
3A2	-	-	-	1	2.23	2.23
**3B**	2	13.57	13.57	2	13.57	13.57
3B1	-	-	-	2	13.57	13.57
3B2	-	-	-		-	-
**3C**	8	23.55	25.84	8	23.55	25.84
3C1	3	31.58	33.39	3	31.58	33.39
3C1i	-	-	-	1	13.47	13.47
3C1ii	-	-	-	2	40.63	43.35
3C2	5	18.73	21.31	5	18.73	21.31
3C2i	-	-	-	-	-	-
3C2ii	--	-	-	5	18.73	21.31
**4**	10	16.20	19.39	10	16.20	19.39
**4A**	-	-	-	-	-	-
**4B**	10	16.20	19.39	7	17.33	20.18
**4C**	-	-	-	3	13.58	17.56

Note: DFS, disease-free survival; OS, overall survival.

## Data Availability

The datasets used or analyzed during the current study are available from the corresponding author, S.S., on reasonable request.

## Abbreviations

The following abbreviations are used in this manuscript:

AIC	Akaike information criterion
ASA	American Society of Anesthesiologists
BIC	Bayesian information criterion
BMI	Body mass index
BSO	Bilateral salping-ooferectomy
CS	Cesarean section
CAD	Coronary artery disease
CT	Chemotherapy
CRT	Chemoradiotherapy
D&C	Dilatation and curretage
DFS	Disease-free survival
ECOG PS	Eastern Cooperative Oncology Group performance status
FIGO	International Federation of Gynecology and Obstetrics
IHC	Immunohistochemistry
LGESS	Low-grade endometrial stromal sarcoma
LVSI	Lymphovascular space invasion
HGESS	High-grade endometrial stromal sarcoma
NVD	Normal vaginal delivery
SD	Standard deviation
UC	Uterine carcinosarcoma
OS	Overall survival
